# Cocaine

**Published:** 1885-03

**Authors:** Sarah Hackett Stevenson

**Affiliations:** Wiesbaden, Germany


### Cocaine.

WIESBADEN; Germany, Dec. 1, 1884.

MR. EDITOR:—E. Merck, the celebrated pharmaceutist of Darmstadt, has kindly furnished me with the following items in regard to the now famous remedy—cocaine:

The chemical formula according to Lossen, is: $C_{17} H_{24} NO_4$. Cocaine is the alkaloid contained in the coca leaves (Erythoxylon Coca Lam) which was first isolated by Niemann. In 1862, Lossen discovered a second principle contained in the same leaves, viz.: hygein, which is of a volatile nature, and has received but little investigation so far; it seems to have a weak and hardly characteristic action. The leaves further contain ecgonin, coca tannin, and a peculiar wax.

The coca crystals belong to the monocline system, melt at 98° C., easily soluble in alcohol, more easily in ether, but only dissolve in 704 parts water. The cocaine *salts*, however, are easily soluble in water.

The first reports of the internal use of coca leaves are handed down from the sixteenth century (Dr. Monardes, Seville, 1569). Its first appearance in Europe dates from 1749—it was described by Jussieu, and was named erythoxylon coca by Lamarck. Tschudi, Markham, Poppig, and others who traveled in South America, found that the natives were in the habit of chewing coca leaves as a remedy for or preventive against the effects of extraordinary exertions. It seems, however, that the Indians chewed the leaves, together with the ashes of chenopodium. Quinea, the alkali in these ashes, probably eliminated the tannin from the coca leaves, leaving free the alkaloid.

Cocaine is believed to be the really active principle of the coca leaves. At first, on apparently good grounds, it was believed that this alkaloid possessed a property analogous to caffeine, theine, and theobromine, viz.: the checking of waste in the body, but Merck declares it impossible to absolutely confirm this theory. Only the other day I saw it in the same list with guarana, caffeine, theine, etc., but was not considered to be as powerful as caffeine!

Cocaine has in small doses an exhilerating effect on the nerve centers, and it takes but a very small dose to produce toxic effects, especially in cold-blooded animals, but fortunately the effects of the drug do not seem to be cumulative. Schroff, who first experimented with cocaine in 1862, observed that 0.05 grm. given internally to rabbits caused great variation in the pulse and respiration, also temporary mydriasis; the same dose, subcutaneously, caused death in convulsions of an epileptic form, also mydriasis in a strong degree, which disappeared at once after death. In frogs 0.001 grm. produced complete loss of motion, preceded by excitement, while a dose of 0.002 grm. proved fatal.

According to Fronmuller, in 1863, a dose of 0.03–0.33 grm. given internally did not have any important effect upon human beings, simply causing sleep in some, accelerated and then retarded pulse and respiration in others. In a case of attempted suicide 1.5 grammes produced no serious injury. Explanatory of this Mr. Merck suggests that the so-called cocaine of that time may not have been really pure cocaine. The preparation with which experiments have been and are being made on this side is Merck's cocaine murias sol. and the dose is 0.05 grm.; however, as much as 0.5 grm. of this solution has been administered subcutaneously. The effects of a subcutaneous injection are first a feeling of warmth, then an insensibility to feeling in the neighborhood of the part, and finally a reddening of the skin. In thirty minutes' time these all disappear.

Dr. Aschenbrandt has found in cocaine an admirable restorative from the exhaustion of diarrhœa, as stated in No. 50 of the *Deutsche Medicin Wochenschr.*, 1883. Prof. Dr. E. V. Fleischel, of Vienna, has proved, to his own satisfaction at least, that cocaine subcutaneously administered is a valuable aid in preventing and curing the morphia habit. (I myself have had excellent results from it with tobacco chewers who were anxious to break the habit.) In America, W. H. Bently observed the good effects of cocaine in overcoming the taste for morphia. Dr. Freud, of the Vienna General Hospital, reports a case of the morphia habit cured within ten days by the use of 0.1 grm. injected subcutaneously three times daily. His theory is that morphia and cocaine are antagonistic. Dr. Freud likewise credits this alkaloid with aphrodisiac properties.

According to the experiments collected by Merck, the therapeutical value of cocaine may be thus described: Cocaine is a stimulant which quickly increases and serves to sustain, in a harmless manner, the physical powers of the body; it is much more strengthening than alcohol—hence its value on all occasions of extraordinary bodily exertion—in doses of 0.05 grm. at intervals. Whether the powers of the mind can likewise be increased and strengthened is still unknown, as also whether the activity of the nerve centers can be permanently increased. Some good results have been obtained in the treatment of melancholia by the subcutaneous method. It is valuable as a stomachic, particularly after a debauch in eating or drinking. It is credited with the permanent cure of atonic indigestion. The various cachexias, phthisis, exhausting fevers, mercurial poisoning, etc., are said to be averted by its use. Its value in dipsomania is not so well established as in the morphia habit, though there are many satisfactory experiments in that direction. As is well known, cocaine paralyzes the sensation of the mucous membrane when brought in contact with the membrane; hence its possible future in diseases of the throat, and in operations about the mouth and throat. (I have not seen any account of experiments of others, but I am making some observations and trials in regard to the use of the drug subcutaneously in perineorrhaphy and trachelorrhaphy. It will be a happy day for surgeons if these minor operations can be done without ether.)

Of its ophthalmological value enough has already been said.

Cocaine citras is used by dentists to anæsthetize the dental nerves. Mr. Merck tells me that this salt can be readily kneaded into pills, which may be wrapped in wadding, moistened and placed in the hollow of the tooth, when lo! the tooth can then be cleaned and filled without pain. If this be true, the man who discovered it is worthy of a high place among the benefactors of the race.

Merck's preparations include the pure cocaine alkaloid, also the salts, viz.: Hydrochlorate, salicylate, hydrobromate, tartrate, and citrate. The experiments mentioned above have been made with the hydrochlorate in about 0.05 gramme doses. Von Hoffman, of Baden-Baden, recommends the salicylate for ophthalmological use, but Merck observes that it still remains to be seen which salt is the best for the eye. Finally, cocaine is not a simple substance. When treated with concentrated hydrochloric acid, it splits up into ecgonin, benzoic acid, and methyl alcohol. Mr. Merck informs me that he hopes to be able ere long to report on the physiological action of ecgonin.

That your readers may possibly be as much interested in this subject as I am, is my only apology for thus intruding upon your valuable space.

Very sincerely yours,

SARAH HACKETT STEVENSON, M. D.

—*Journal of American Medical Association.*

